# Distinct functional subnetworks of cognitive domains in older adults with minor cognitive deficits

**DOI:** 10.1093/braincomms/fcae048

**Published:** 2024-02-15

**Authors:** Nadieh Drenth, Suzanne E van Dijk, Jessica C Foster-Dingley, Anne Suzanne Bertens, Nathaly Rius Ottenheim, Roos C van der Mast, Serge A R B Rombouts, Sanneke van Rooden, Jeroen van der Grond

**Affiliations:** Department of Radiology, Leiden University Medical Center, P.O. Box 9600, 2300 RC Leiden, The Netherlands; Department of Radiology, Leiden University Medical Center, P.O. Box 9600, 2300 RC Leiden, The Netherlands; Department of Radiology, Leiden University Medical Center, P.O. Box 9600, 2300 RC Leiden, The Netherlands; Department of Psychiatry, Leiden University Medical Center, P.O. Box 9600, 2300 RC Leiden, The Netherlands; Department of Radiology, Leiden University Medical Center, P.O. Box 9600, 2300 RC Leiden, The Netherlands; Department of Psychiatry, Leiden University Medical Center, P.O. Box 9600, 2300 RC Leiden, The Netherlands; Department of Psychiatry, Leiden University Medical Center, P.O. Box 9600, 2300 RC Leiden, The Netherlands; Department of Psychiatry, Leiden University Medical Center, P.O. Box 9600, 2300 RC Leiden, The Netherlands; Department of Psychiatry, Collaborative Antwerp Psychiatric Research Institute (CAPRI)–University of Antwerp, Antwerp, Belgium; Department of Radiology, Leiden University Medical Center, P.O. Box 9600, 2300 RC Leiden, The Netherlands; Institute of Psychology, Leiden University, P.O. Box 9555, 2300 RB Leiden, The Netherlands; Leiden Institute for Brain and Cognition, P.O. Box 9600, 2300 RC Leiden, The Netherlands; Department of Radiology, Leiden University Medical Center, P.O. Box 9600, 2300 RC Leiden, The Netherlands; Department of Radiology, Leiden University Medical Center, P.O. Box 9600, 2300 RC Leiden, The Netherlands

**Keywords:** functional connectivity, resting-state networks, cognitive domains, older adults, functional MRI

## Abstract

Although past research has established a relationship between functional connectivity and cognitive function, less is known about which cognitive domains are associated with which specific functional networks. This study investigated associations between functional connectivity and global cognitive function and performance in the domains of memory, executive function and psychomotor speed in 166 older adults aged 75–91 years (mean = 80.3 ± 3.8) with minor cognitive deficits (Mini-Mental State Examination scores between 21 and 27). Functional connectivity was assessed within 10 standard large-scale resting-state networks and on a finer spatial resolution between 300 nodes in a functional connectivity matrix. No domain-specific associations with mean functional connectivity within large-scale resting-state networks were found. Node-level analysis revealed that associations between functional connectivity and cognitive performance differed across cognitive functions in strength, location and direction. Specific subnetworks of functional connections were found for each cognitive domain in which higher connectivity between some nodes but lower connectivity between other nodes were related to better cognitive performance. Our findings add to a growing body of literature showing differential sensitivity of functional connections to specific cognitive functions and may be a valuable resource for hypothesis generation of future studies aiming to investigate specific cognitive dysfunction with resting-state functional connectivity in people with beginning cognitive deficits.

## Teaser

Drenth *et al*. investigated associations between functional connectivity and cognition in 166 non-demented older adults with minor cognitive deficits. The authors show that cognitive performance is differentially associated with functional connectivity per cognitive domain. Distinct subnetworks of functional connections were identified related to memory, executive function and psychomotor speed performance.

## Graphical Abstract

**Graphical Abstract:**
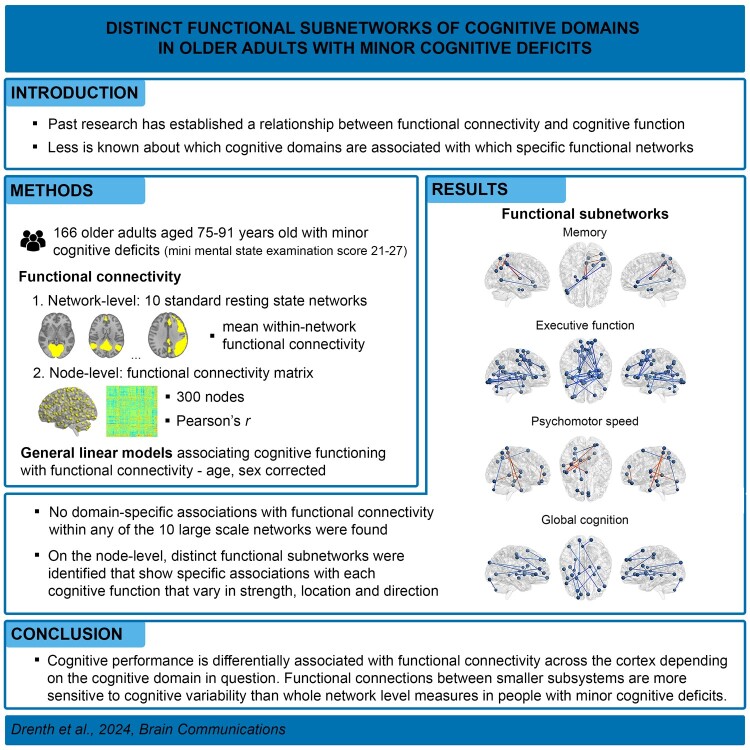

## Introduction

Over the past decades, it has become clear that cognitive functions are not localized in specific brain regions but rather are supported by the connections between brain regions. These functional networks of connected brain regions have been related to numerous behavioural and cognitive functions.^1,2^ Alterations in the connectivity of functional networks are related to cognitive dysfunction both in normal aging^3-5^ and in neurodegenerative disease.^6,7^ Aberrant functional connectivity patterns can already be identified in early stages of cognitive impairment, such as in individuals experiencing subjective cognitive decline (SCD) or mild cognitive impairment (MCI).^8-11^ Moreover, functional connectivity patterns are predictive of future cognitive decline.^12-14^

Recent evidence suggests variation exists in the association between functional connectivity and cognition depending on distinct cognitive domains.^15,16^ Nevertheless, it remains unclear which specific cognitive domains are associated with which specific functional (sub)networks. More knowledge on the specificity of the functional connectivity–cognition relationship may provide further insight into mechanisms underlying diseases associated with cognitive deterioration. Furthermore, it may inform hypothesis generation about functional connectivity alterations in people with specific cognitive deficits and increase sensitivity of studies by helping to focus the research onto relevant (sub)networks of functional connections.

This study aimed to investigate whether distinct cognitive functions were differentially associated with functional connectivity, both in location and in degree. To this end, we examined in a large sample of non-demented older adults with minor cognitive deficits whether global cognitive function and performance in the domains of memory, executive function and psychomotor speed were associated with functional connectivity within validated standardized large-scale brain networks and in smaller subnetworks of connectivity.

## Materials and methods

### Participants and procedures

Participants were 166 community-dwelling older adults with minor cognitive deficits that participated in an earlier study.^17^ All participants were 75 years or older, had hypertension that was medication controlled with a current systolic blood pressure ≤ 160 mmHg and a Mini-Mental State Examination (MMSE) score between 21 and 27. Exclusion criteria included a history of stroke or transient ischaemic attack, major cardiovascular disease, including heart failure, or a clinical diagnosis of dementia. The study was approved by the Medical Ethical Committee of the Leiden University Medical Center, and written informed consent was obtained from all participants.

Participants underwent MRI and neuropsychological testing. We included participants in the present analysis if they had completed a resting-state functional MRI (RS-fMRI) scan and the neuropsychological testing (*n* = 202). Of those, 36 participants were excluded due to suboptimal quality of RS-fMRI (corrupted files *n* = 4; lack of whole brain coverage *n* = 12; or excessive motion, i.e. ≥3 mm movement in any direction, *n* = 20), resulting in a sample of 166 participants for the present investigation.

### Data acquisition and preprocessing

Participants were scanned at the Leiden University Medical Center on a 3 Tesla Philips Achieva MRI Scanner (Philips Medical Systems, Best, The Netherlands) equipped with a standard 32-channel head coil. RS-fMRI was acquired with echo planar imaging with repetition time (TR) = 2200 ms, echo time (TE) = 30 ms, flip angle (FA) = 80°, field of view (FOV) = 220 × 220 × 113 mm, 38 slices with 10% interslice gap, resulting in a voxel size of 2.75 × 2.75 × 2.72 mm and total scan duration of 7 min 29 s. Additionally, a 3D T_1_-weighted (3D-T_1_) structural image was acquired with TR = 9.7 ms, TE = 4.6 ms, FA = 8°, FOV = 224 × 177 × 168 mm and voxel size = 1.17 × 1.17 × 1.40 mm. Preprocessing of RS-fMRI and 3D-T_1_ images was performed with FMRIB’s Software Library (FSL, version 6.0.4^18^) and included brain extraction,^19^ denoising of the data using motion correction with MCFLIRT,^20^ spatial smoothing at full width at half maximum of 5 mm and ICA-AROMA denoising.^21,22^ Further, a high-pass temporal filter at 0.01 Hz was applied to remove very low-frequency drifts from the data. Lastly, spatial normalization to the MNI152 2 mm template (Montreal Neurological Institute, Montreal, QC, Canada) using FLIRT^20,23^ and FNIRT^24^ with a 10 mm warp was performed.

### Functional connectivity

Functional connectivity was assessed on both a network level and a node level. On the network level, standard resting-state network (RSN) templates were used. A network refers to a set of brain regions that are functionally connected, that is: the resting-state signal fluctuations of these brain regions are temporally correlated. On the node level, a graph theoretical approach was used where a fine functional parcellation divided the brain into 300 brain regions, or so-called nodes. Pearson’s correlation coefficient was calculated between all nodes to determine the functional connectivity between them.

**Figure 1:**
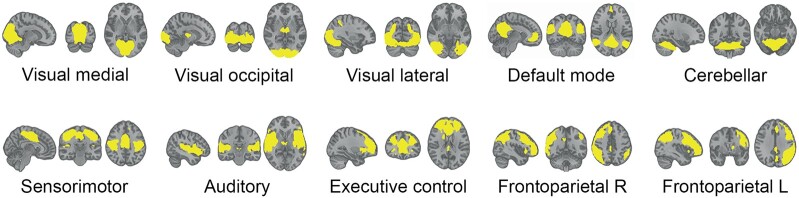
**Ten standard RSNs.** Standard RSNs^1^ are superimposed on the MNI standard anatomical brain image. R, right; L, left.

Large-scale brain networks can be consistently and reliably identified across subjects^1,25,26^ using an independent component analysis (ICA) approach. Because of the nature of ICA, the precise decomposition of the networks can vary each time the algorithm is run, resulting in slightly different networks, which can hamper the generalizability of the results and comparability to other studies. To overcome this inherent constraint, standardized network templates can be used to ensure reliable and consistent decomposition of the networks. We chose to use the validated standardized network templates provided by Smith *et al*.^1^ to investigate functional connectivity within the following 10 common networks: the visual medial network, visual occipital network, visual lateral network, default mode network, cerebellar network, sensorimotor network, auditory network, executive control network, frontoparietal right network and frontoparietal left network (Fig. 1). The functional (i.e. behavioural) interpretation of these RSNs has been thoroughly investigated,^2^ and the RSNs are highly similar to task-activated networks,^1^ showing that this set of RSNs reflects meaningful neurocognitive networks. A dual regression approach implemented in FSL^27,28^ was used to obtain subject-specific time series and spatial maps for each network. A white matter and CSF template were included for nuisance regression. Mean functional connectivity within each network was extracted (*Z*-score) from each subject’s spatial maps of each network where higher scores indicated higher within-network functional connectivity.

Second, functional connectivity was determined on a finer level using a graph theoretical approach. Here, the brain network is defined as a graph consisting of nodes and edges where nodes represent the elements of the network (i.e. small brain regions) and edges represent the interaction (in this case the functional connectivity) between the nodes. A total of 300 nodes were defined based on the functional parcellation of Seitzman *et al*.^29^ by drawing spheres around the provided MNI coordinates resulting in 239 cortical nodes (5 mm radius), 34 subcortical nodes (4 mm radius) and 27 cerebellar nodes (4 mm radius). Subcortical and cerebellar nodes have a smaller radius than cortical nodes to correspond better to the functional boundaries of these smaller anatomical regions. To calculate the edges, mean time series of all 300 nodes were extracted from subjects’ preprocessed RS-fMRI scans, and Pearson’s correlation coefficients were calculated between the mean time series of all nodes with each other. Nuisance regression was performed prior to time series extraction where mean time series of CSF and white matter were regressed out of the data. Using GraphVar (version 2.03a)^30^ implemented in MATLAB (version 2020b), a functional connectivity matrix was constructed for each subject where rows and columns represent the nodes and matrix values represent the correlation coefficients (edges) between the nodes. This resulted in a matrix of 300 × 300 that contained 44 850 unique connectivity values ((*N*(*N* − 1))/2) for each subject. Matrices were Fisher’s *R*- to *Z*-transformed.

### Cognitive function

Global cognitive function was assessed with the MMSE (range 0–30), where higher scores indicated better performance.^31^ Further, a battery of cognitive tests was administered from which three cognitive domain scores were calculated. Executive function was assessed with the difference between the time to complete the Trail Making Test (TMT) A and B^32^ and the interference score of the abbreviated Stroop Color–Word Test.^33^ Scores of both executive tests were reversed so that higher scores indicated better performance. Memory was assessed with the Visual Association Test^34^ (range 0–12) and the immediate (three trials, scoring range 0–45) and delayed recall (one trial, scoring range 0–15) of the 15-Word Verbal Learning Test.^34^ Psychomotor speed was measured by the number of correctly coded digits after 90 s of the Letter Digit Substitution Test^35^ and the time to complete the TMT-A (reverse scored). Compound scores were calculated by first converting raw test scores into standardized *Z*-scores (($x-\;\bar{x}$)/SD) and then calculating the mean *Z*-score for all tests included in the domain score.^17^

### Statistical analysis

Associations between cognitive scores and functional connectivity were assessed for both functional connectivity measures. For each network, linear regression models were constructed using SPSS (version 29.0) to assess if performance in the cognitive domains were associated with functional connectivity in the large-scale RSNs. Mean functional connectivity within the RSN was the dependent variable, the cognitive domain scores of memory, executive function and psychomotor speed were added as independent variables, and age and sex were added as control variables. Linear regression models that included the MMSE score as independent variable were calculated separately from the cognitive domain scores to avoid entering overlapping variables in the same model. All variables were checked for normality beforehand and transformed using a natural log transformation if needed. Model assumptions were checked, and outliers were removed if necessary. The significance threshold was Bonferroni adjusted for 10 RSNs and set to 0.005.

Second, to assess whether there are localized connections that are associated with specific cognitive functions, edgewise statistical analyses were performed on the functional connectivity matrix using GraphVar. General linear models with 10 000 permutations were constructed where the functional connectivity between any two nodes was the dependent variable, the cognitive domain score was the independent variable, and age and sex were added as control variables. To correct for the large number of multiple comparisons (44 850 edges), a false discovery rate correction was applied, restricting type I errors. Then, the ‘GetComponent’ feature of GraphVar was used to extract components (i.e. subnetworks consisting of subsets of nodes and edges) related to the cognitive domain tested. This showed which nodes and edges were related to performance on the cognitive domain of interest.

Additionally, canonical correlation analysis was used to explore potential multivariate associations between cognitive function and functional connectivity. Canonical correlation analysis searches for linear combinations between two sets of variables. The three cognitive domain scores were defined as the first set of variables, and the mean functional connectivity within each of the 10 RSNs were defined as the second set of variables. Subsequently, a second canonical correlation analysis was conducted focusing on local functional connections. For this, a principal component analysis was performed on the functional connectivity matrix to reduce the dimensionality of the data. The top components that accounted for >80% of the total variance were selected, resulting in 100 components that explained 81% of the total variance. These components were utilized as the second set of variables in the canonical correlation analysis. The significance threshold for all canonical correlations was set at 0.05.

## Results

Table 1 describes the demographic characteristics of the study sample. On average, participants were 80 years old and experienced mild to moderate cognitive deficits.

**Table 1:** Characteristics of the study population (*n* = 166)

Characteristics	
**Demographic and clinical**	
Age, years	80.3 (3.8)
Sex, female	96 (57.8%)
>6 years of education^a^	109 (65.7%)
Hypertension	166 (100%)
Systolic blood pressure, mmHg	145.2 (21.9)
Diastolic blood pressure, mmHg	80.7 (11.2)
**Cognition**	
MMSE	26 (25–27)
ΔTMT, s^b^	133.8 (66.4)
Stroop interference score, s^b^	241.8 (228.3–251.6)
VAT, pictures remembered	12 (10–12)
WVLT immediate, words remembered	16.9 (5.4)
WVLT delayed, words remembered	4.6 (2.6)
LDST, digits coded	30.8 (9.1)
TMT-A, s^b^	244.0 (225.8–257.0)

Data are presented as mean (SD), median (interquartile range) or number (percentage) where appropriate. MMSE, Mini-Mental State Examination (range 0–30); TMT, Trail Making Test; VAT, Visual Association Test (range 0–12); WVLT, 15-Word Verbal Learning Test (immediate range 0–45, delayed range 0–15); LDST, Letter Digit Substitution Test.

^a^Missing for *n* = 10 participants.

^b^Reverse scored so that higher scores indicate better performance.

Each of the standard RSNs is displayed in Fig. 1. Linear regression models were used to assess associations between cognitive performance in any of the domains or global cognitive function with functional connectivity within each RSN. No significant associations were found for any of the three cognitive domains with functional connectivity within any of the RSNs. For global cognitive function, only the association with functional connectivity within the cerebellar network was significant (*β* = −0.222, *P* = 0.004). No multivariate associations between cognition and functional connectivity within the networks were identified as none of the canonical functions were significant (*λ* = 0.76, *F* = 1.45, *P* = 0.060; *λ* = 0.91, *F* = 0.80, *P* = 0.696; and *λ* = 0.98, *F* = 0.46, *P* = 0.885).

**Figure 2:**
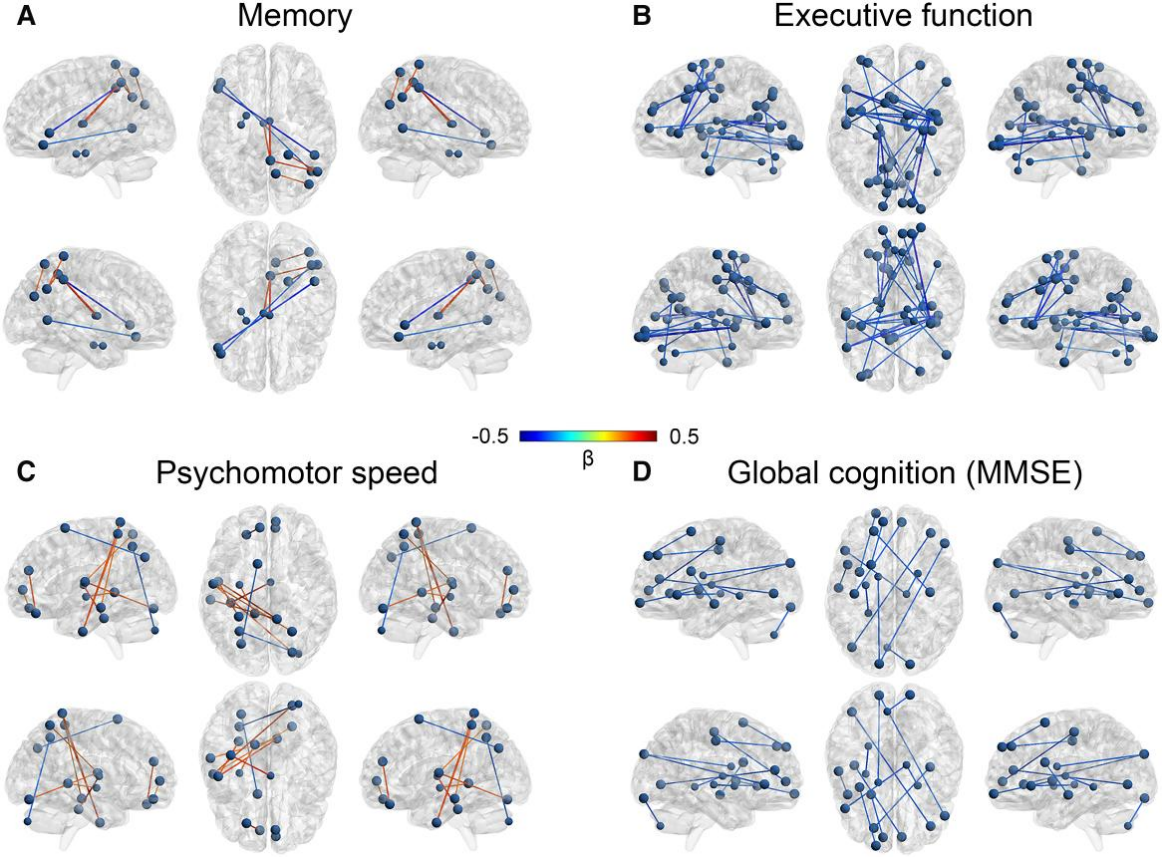
**Functional subnetworks per cognitive domain.** Each panel displays the subnetworks of functional connectivity that are significantly associated with cognitive performance in the domain of (A) memory, (B) executive function, (C) psychomotor speed, and (D) global cognition, as identified with a general linear model (controlled for age and sex) in *n* = 166 with a false discovery rate correction using GraphVar.^30^ Lines between nodes show standardized regression weights, and line colours indicate whether the association between cognitive test scores and functional connectivity of the connected nodes is negative (blue) or positive (red). Negative associations indicate that lower cognitive performance is associated with higher functional connectivity between nodes. Positive associations indicate that higher cognitive performance is associated with higher functional connectivity between nodes. Brain networks were visualized with the BrainNet Viewer (http://www.nitrc.org/projects/bnv/).^36^

Edgewise analysis on the functional connectivity matrix revealed several subnetworks where functional connectivity was associated with the severity of cognitive deficits (controlled for age and sex). Figure 2 shows which subnetworks were significantly related to each of the cognitive domain and global cognition (MMSE) scores with a colour scale indicating the direction and strength of the cognition–connectivity associations.

Memory performance showed both negative and positive cognition–connectivity associations (Fig. 2A). Memory performance was positively associated with connectivity between nodes in the parietal cortex (right superior lateral occipital cortex), as well as between right parietal (superior parietal lobule, angular gyrus and precuneus) and subcortical (left and right thalamus) areas. Negative associations were found between the left frontal orbital cortex and right temporooccipital middle temporal gyrus, left inferior frontal gyrus pars triangularis and right posterior supramarginal gyrus and between the left amygdala and left hippocampus.

Executive function involved all cortices and showed cognition–connectivity sensitivity that can roughly be divided into a frontal cluster and posterior inferior cluster (Fig. 2B). All subnetworks showed negative associations, indicating lower executive function performance was associated with higher connectivity in these subnetworks. A frontal subnetwork was found that connected bilateral precentral gyri, right superior frontal gyrus, bilateral frontal pole, left paracingulate gyrus and left inferior frontal gyrus pars triangularis with each other. Other subnetworks primarily connected posterior brain regions with each other. Left intrahemispheric subnetworks connected the thalamus, parietal (posterior cingulate gyrus, precuneus and superior lateral occipital cortex) and occipital (intracalcarine cortex) areas, as well as frontal pole and posterior cingulate gyrus. Right intrahemispheric subnetworks showed connections between the amygdala, thalamus and crus I of the cerebellum, between the hippocampus and cerebellar vermis VI, between the precuneus and area VIIb of the cerebellum and between the thalamus and inferior frontal gyrus pars triangularis. Additionally, interhemispheric connections were found where the right insula connected to the left precuneus, right occipital fusiform gyrus and bilateral occipital pole, the left thalamus to the left anterior inferior temporal gyrus, left insula and right occipital pole, and the right precentral gyrus to the left anterior cingulate gyrus.

Subnetworks sensitive to the psychomotor speed domain primarily consisted of parietal and subcortical/temporal nodes (Fig. 2C). Positive associations between cognitive scores and functional connectivity were found for most subnetworks. A temporal–parietal subnetwork was identified consisting of the left posterior and anterior inferior temporal gyri, left superior parietal lobule and right postcentral gyrus. Additionally, a parietal–occipital subnetwork connecting the left central opercular cortex to the right superior lateral occipital cortex and the right occipital fusiform gyrus was found. Further, frontal connections in the right hemisphere between the paracingulate gyrus and the frontal medial cortex and in the left hemisphere between the frontal pole and the paracingulate gyrus were identified. Subcortical areas also showed positive cognition–connectivity associations between the left putamen and the left hippocampus and the right thalamus with the left posterior temporal fusiform cortex. Negative associations were found for the left superior lateral occipital cortex and left superior frontal gyrus, as well as the right cerebellar crus II and left superior parietal lobule.

Global cognitive functioning, as measured by the MMSE, showed all negative associations with functional connectivity involving mostly frontal brain regions (Fig. 2D). Subnetworks connectingthe right thalamus to the right putamen and left inferior frontal gyrus pars triangularis, and the left occipital pole connecting the left caudate and right inferior frontal gyrus pars triangularis were identified. Additionally, the left frontal pole connected to the left insula, left superior frontal gyrus and right precentral gyrus, and the left precentral gyrus to the right paracingulate gyrus. The right frontal pole connected to the left inferior lateral occipital cortex and the right occipital fusiform gyrus to left VIIb of the cerebellum. Left intrahemispheric subnetworks were found connecting the putamen and the hippocampus, as well as the inferior frontal gyrus pars opercularis and the posterior middle temporal gyrus.

No multivariate associations between cognition and local functional connections were identified as none of the canonical functions were significant (*λ* = 0.05, *F* = 1.06, *P* = 0.328; *λ* = 0.16, *F* = 0.96, *P* = 0.613; and *λ* = 0.42, *F* = 0.93, *P* = 0.625).

## Discussion

This study aimed to investigate the association between functional connectivity and cognition for distinct cognitive functions. For global cognitive function and the cognitive domains of memory, executive function and psychomotor speed, we examined associations between cognitive performance and functional connectivity on a network level as well as on a node level in older adults with minor cognitive deficits. Our data show that cognitive performance was not associated with functional connectivity within large-scale brain networks, but on a node level, differences in strength, direction and location of associations between functional connectivity and cognition were found for global cognitive function and specific cognitive domains.

Aberrant functional connectivity patterns can already be identified early in the spectrum of cognitive decline, such as in individuals experiencing subjective cognitive complaints or MCI.^8-11^ In this study, we did not find direct associations between mean functional connectivity within 10 large-scale standard networks and global cognitive performance or cognitive performance in the domains of memory, executive function and psychomotor speed in a large group of older adults with minor cognitive deficits. The association between global cognition and functional connectivity within the cerebellar network reached significance; however, we consider this to be a coincidence, not reflecting a true effect given the borderline significance and, most importantly, the finding is not reflected in the node-level analyses where global cognition is barely associated with the cerebellum. Finding no association between cognition and within-network functional connectivity might be a consequence of the coarse scale of this functional connectivity measure and of the fact that our study sample experienced only minor cognitive deficits. Larger effects detectable on a whole network level might be more likely to occur in case of more severe cognitive deficits.^7^ It is possible that associations in this group might be too small or too localized to be detected at the whole network level or that conflicting effects may occur within the network. To illustrate, in patients with mild cognitive deficits, both hyper- and hypo-connectivity within the default mode network may occur,^9^ which might lead to a cancellation effect on the whole network level. Our data show that mean functional connectivity within large-scale networks does not appear to be a very sensitive measure to investigate the relationship between functional connectivity and cognitive dysfunction in people experiencing minor cognitive deficits.

What seems more informative is functional connectivity on a finer spatial scale. In line with recent literature,^15,16^ our node-level analyses revealed different associations between functional connectivity and cognitive performance across different cognitive domains. Associations differed in strength, direction and location with different subnetworks involved for individual cognitive domains. Memory performance showed significant associations with functional connectivity between brain regions belonging mainly to the default mode and frontoparietal networks, which are networks that have previously been related to memory function.^1,2,15^ Interestingly, our data showed both positive and negative associations, indicating that both higher and lower functional connectivity were associated with better memory performance. Functional connections between parietal and subcortical regions (precuneus, thalamus) showed positive associations with memory performance, while functional connections between subcortical (amygdala, hippocampus) and frontoparietal regions (orbitofrontal cortex, middle temporal and supramarginal gyri) showed negative associations with memory performance. Supported by recent findings from Dautricourt *et al.*,^13^ who also found both positive and negative relationships of memory performance with functional connectivity between the same brain regions, our findings indicate that these specific functional connections are particularly important for memory function and both higher and lower functional connectivity may favour memory function depending on the specific functional connection. Psychomotor speed function involved mainly functional connections between sensory networks (visual, auditory and sensorimotor networks) and the executive control network, which is not surprising considering psychomotor tasks require sensory resources as well as some executive control, for example interference control. Additionally, a few nodes belonging to default mode and frontoparietal networks showed an association with psychomotor speed, similar to Shaw *et al*.^15^ For the cognitive domain of executive function, a widespread collection of functional connections related to executive performance was found. Functional connections between areas of sensorimotor and visual (medial and occipital) networks, bilateral frontoparietal networks, executive control network and default mode network were related to executive function performance. Functional connectivity between these brain regions has been implicated in executive control function in patients with MCI and Alzheimer’s disease.^8,37,38^ These findings show that many subsystems of large-scale networks are important for executive performance and reflect the complexity of executive functions requiring cognitive resources as well as motor and visual systems. Likewise, global cognitive function (MMSE) also involved a mixture of networks, mostly functional connections between frontoparietal and visual networks, and executive control and sensorimotor networks. In line with our network-level results, these findings indicate that mainly interactions between networks rather than within networks seem important for cognitive functioning, where specific functional connections have different sensitivities for specific cognitive domains. Alterations in between-network connectivity have previously been identified in people with cognitive dysfunction.^39-41^ For example, functional connectivity between frontoparietal and default mode networks has been related to memory function in older adults across the Alzheimer’s disease spectrum.^39^ Our findings further highlight the importance of functional connectivity between (sub)networks for cognitive functioning in people with minor cognitive deficits.

An implication of our findings might be that cognitive status (severity of deficits and specific domains affected) of study samples may be a potentially important confounder in functional connectivity research in people with beginning cognitive deficits. The literature on functional connectivity alterations in people with minor cognitive deficits is relatively inconsistent. Our findings suggest that cognitive status might play a role in the between-study variability in functional connectivity in SCD and MCI literature. Recently, Eyler *et al.*^9^ investigated whether variation in the literature of default mode network functional connectivity alterations in people with MCI could be explained by sample characteristics or methodological differences between studies. Neither sample characteristics (age and gender) nor methodological differences (e.g. seed based, ICA or other functional connectivity methodology) could explain variation between studies.^9^ Differences in cognitive status of the study samples were not considered; however, our results indicate that variation in cognitive performance between individuals or groups may result in different functional connectivity–cognition relationships. This is supported by Mueller *et al*.^42^ who showed that brain regions that show high intersubject variability in functional connectivity are predictive of behavioural differences between subjects. Nonetheless, we do not expect cognitive status to fully explain functional connectivity variability between studies in SCD and MCI, but it may contribute to explaining smaller differences in findings between studies as our results showed differences in smaller subsystems depending on cognitive performance and domains affected.

Strengths of our study include the use of multilevel measures of both functional connectivity and cognition. We took an exploratory approach that covered multiple networks and multiple cognitive domains. Further exploration opportunities might lie in examining the association across the frequency range of the resting-state fMRI signal. Although resting-state functional connectivity is predominantly thought to reside in the lower frequencies, the precise role of the higher frequencies in functional connectivity is still under investigation. It has been shown that higher frequencies contain connectivity information^43^ that is also behaviourally relevant.^44^ What is more, connectivity strength and network organizational properties might differ across the frequency range.^45,46^ Contradictory evidence exists regarding spatial patterns of functional networks, where some results suggest that functional connectivity patterns are similar across the frequency range^43,45^ while other findings indicate compositional differences between frequency bands.^47,48^ While our study was underpowered to properly investigate frequency subbands and multiband acquisition protocols are preferred, future studies could investigate whether the associations between functional connectivity and distinct cognitive functions differ across the frequency spectrum and what the behavioural consequences of these differences might be to further improve our understanding of brain function in health and disease.

In conclusion, we showed that cognitive performance is differentially associated with functional connectivity across the cortex depending on the cognitive domain in question. Functional connections between smaller subsystems are more sensitive to cognitive variability than whole network-level measures in individuals with minor cognitive deficits. Therefore, an implication for future research seems to be that methods with a finer spatial resolution, such as node-level analyses, are preferable over average network-level measures of functional connectivity when investigating the relationship between functional connectivity and cognitive functioning in early stages of cognitive decline. Furthermore, cognitive status, that is which cognitive domains are affected and to what extent, may be an important between-subject variable to consider in order to improve our understanding of functional connectivity patterns in people with minor cognitive deficits. Our findings add to a growing body of literature showing differential sensitivity of functional connections to specific cognitive functions. The results can be useful for hypothesis generation of future studies that aim to investigate specific cognitive dysfunction with resting-state functional connectivity in people with beginning cognitive deficits.

## Data Availability

The data that support the findings of this study are available from the corresponding author, upon reasonable request.
